# Heterogeneity in Unmet Treatment Need and Barriers to Accessing Mental Health Services Among U.S. Military Service Members with Serious Psychological Distress

**DOI:** 10.1007/s10488-023-01289-4

**Published:** 2023-08-18

**Authors:** Michael S. Dunbar, Joshua Breslau, Rebecca Collins, Robin Beckman, Charles C. Engel

**Affiliations:** 1 RAND Corporation, 4750 Fifth Avenue, Suite 600, Pittsburgh, PA 15213-2665 USA; 2 RAND Corporation, 1776 Main Street, Santa Monica, CA 90407 USA; 3 Department of Psychiatry and Behavioral Sciences, University of Washington, 1959 NE Pacific Street, Seattle, WA 98195 USA; 4 Health Services Research & Development Center for Innovation, VA Puget Sound Health Care System, 1660 S. Columbian Way, Seattle, WA 98108 USA

**Keywords:** Military, Mental health treatment, Unmet treatment need, Treatment barriers, Psychological distress

## Abstract

The goal of the current study is to examine heterogeneity in mental health treatment utilization, perceived unmet treatment need, and barriers to accessing care among U.S. military members with probable need for treatment. Using data from the 2018 Department of Defense Health Related Behavior Survey, we examined a subsample of 2,336 respondents with serious psychological distress (SPD; past-year K6 score ≥ 13) and defined four mutually exclusive groups based on past-year mental health treatment (treated, untreated) and self-perceived unmet treatment need (recognized, unrecognized). We used chi-square tests and adjusted regression models to compare groups on sociodemographic factors, impairment (K6 score; lost work days), and endorsement of treatment barriers. Approximately 43% of respondents with SPD reported past-year treatment and no unmet need (*Needs Met*). The remainder (57%) met criteria for unmet need: 18% endorsed treatment and recognized unmet need (*Treated/Additional Need*); 7% reported no treatment and recognized unmet need (*Untreated/Recognized Need*); and 32% reported no treatment and no unmet need (*Untreated/Unrecognized Need*). Compared to other groups, those with *Untreated/Unrecognized Need* tended to be younger (ages 18–24; *p* = 0.0002) and never married (*p* = 0.003). The *Treated/Additional Need* and *Untreated/Recognized Need* groups showed similar patterns of treatment barrier endorsement, whereas the *Untreated/Unrecognized Need* group endorsed nearly all barriers at lower rates. Different strategies may be needed to increase appropriate mental health service use among different subgroups of service members with unmet treatment need, particularly those who may not self-perceive need for treatment.

## Introduction

Mental health conditions are a common, serious, and stigmatized problem within the U.S. military. For example, prevalence rates of current major depression and generalized anxiety disorder among military personnel are estimated at 9% and 14%, respectively (Meadows et al., 2018). Serious psychological distress (i.e., symptoms that are severe enough that they are clinically significant and likely to result in functional impairments) (Kessler et al., 2003) among service members may limit service members’ capacity to perform their duties, which has consequences for military readiness (Pflanz, 1999; Pflanz & Ogle, 2006). Mental health conditions are significant contributors to lost work days among active duty service members (Dunbar et al., 2022), leading causes of health care use (including hospitalization) and disability compensation for active duty servicemembers and veterans (Cohen et al., 2010; Hoge et al., 2005; Possemato et al., 2010), and may affect career longevity via impacts on separation from active duty (Garcia et al., 2015; Hoge et al., 2005; Rowan et al., 2014) and retention rates following an initial term of service (Schmied et al., 2012). For individuals experiencing a mental health problem, mental health treatment can reduce occupational impairments (e.g., lost work) and improve work satisfaction, performance and productivity among individuals with mental health conditions like depression (Finkelstein et al., 1996; Mintz et al., 1992; Simon et al., 2000). This has important ramifications for the military’s capacity to maintain a healthy and ready force and underscores a need for ongoing efforts to enhance accessibility and use of appropriate services for those individuals who need them (Hoge et al., 2016).

In the civilian population, approximately one-in-four adults with mental illness perceives some unmet need for treatment (Substance Abuse and Mental Health Services Administration, 2019). Similarly, despite recent trends toward greater utilization of mental health services, unmet need for mental health care among service members remains a serious concern (Fikretoglu et al., 2022; Hom et al., 2017; Quartana et al., 2014). In both civilian and military populations, common barriers including mental health stigma and practical barriers (e.g., scheduling challenges) may limit use of mental health services, even when such services are available (Fikretoglu et al., 2022; Hom et al., 2017; Kim et al., 2011; Mojtabai et al., 2011; Quartana et al., 2014; Tanielian et al., 2016). Service members may also face barriers that are specific to the military context, including beliefs that seeking mental health treatment may have a negative impact on one’s military career (Hoge et al., 2004; Hom et al., 2017; Stecker et al., 2013; Tanielian et al., 2016; Vogt, 2011; Zinzow et al., 2013). In addition, beliefs that one can or should handle the problem on one’s own and/or that one does not need care rank among the most commonly cited barriers to seeking treatment among military servicemembers (Fikretoglu et al., 2022; Hom et al., 2017; Momen et al., 2012; Zinzow et al., 2013).

Recognition of a problem and/or need for treatment is viewed as a key driver of treatment seeking and behavior change/symptom resolution among individuals with psychological disorders (Prochaska & DiClemente, 1992; Prochaska et al., 2015). Consistent with this, one recent study examining mental health and help-seeking among military personnel in the United Kingdom found that most individuals who perceived having a problem with their mental health sought some form of help (Stevelink et al., 2019). Qualitative research with service members also indicates that perceptions of having reached a “crisis point” at which symptoms can no longer be ignored may be an important driver of decisions to seek help (Murphy et al., 2014). Similarly, recognizing more severe or impairing symptoms is associated with mental health utilization among U.S. service members (McKibben et al., 2013). These factors have important implications for efforts to reduce unmet treatment need and its associated negative consequences among military personnel. For example, individuals who do not engage in treatment but recognize a need may experience different barriers to care relative to those without such insight into treatment needs, which has implications for targeted efforts to increase service use among subgroups of individuals who could benefit from treatment.

The U.S. military employs a variety of strategies to address unmet treatment need–such as mental health literacy interventions and mental health stigma reduction campaigns (Acosta et al., 2014, 2016)—that may differentially affect subgroups of service members with and without a perceived need for care. For example, strategies such as mental health literacy training can help to increase awareness of signs and symptoms of mental health problems and effective treatments, which could enhance service use among individuals with serious psychological distress who may not recognize that they could benefit from care. Other strategies aim to reduce barriers like mental health stigma and related career concerns (Acosta et al., 2016), which may be particularly beneficial for individuals who perceive a need for treatment but do not engage in services due to such attitudes (Britt et al., 2016). In addition, ongoing efforts to monitor and improve the quality of mental health care (Hepner et al., 2017) may be instrumental to ensure that the needs of individuals who already access mental health treatment are sufficiently met. However, little is known about the potential targets of these diverse strategies, including whether and how subgroups of individuals with probable need for treatment may differ in relation to size, perceptions of need, and barriers to accessing care.

The goal of the current study is to examine heterogeneity in unmet need for mental health treatment among US military members with high levels of psychological distress. Specifically, we assess differences in a range of individual-level factors—including demographic and service characteristics, lost work, and severity of psychological distress—across the following groups of individuals with need for mental health treatment: (1) individuals who have received mental health treatment and do not report unmet need (i.e., those whose treatment needs were plausibly ‘met’), (2) individuals who received mental health treatment and report residual unmet treatment need, (3) individuals who did not receive mental health treatment and report unmet treatment need, and (4) individuals who did not receive mental health treatment and do not report unmet treatment need. Among service members in groups 2–4, those meeting criteria for *unmet* MH treatment need, we examine differences in types of treatment barriers endorsed.

## Method

Data are from the Active Component of the 2018 Health Related Behaviors Survey (HRBS) (Meadows et al., 2021). The HRBS is large survey of military personnel fielded every two to four years that is sponsored by the U.S. Department of Defense. The HRBS assesses a range of mental and physical health outcomes and related factors, including health-related behaviors, treatment utilization, and other factors that may affect force readiness or military functioning. A stratified random sampling frame (by service branch, pay grade, and gender) was used to ensure representation across key demographic and service groups. In addition, non-response and design weights were applied to help ensure sample representativeness of the active duty service member population. A total of 199,996 individuals were invited to participate and 17,166 completed surveys (weighted response rate of 9.6% for the Active Component) (Meadows et al., 2021). Participation was voluntary and respondents did not receive compensation. In this study, the analytic sample was the subgroup of 2,336 respondents in the Army, Navy, Marine Corps, Air Force (Department of Defense) and the U.S. Coast Guard (Department of Homeland Security) who met study criteria for serious psychological distress in the past year.

### Serious Psychological Distress

Mental health symptoms were assessed using the K6 scale, a reliable, valid 6-item Likert measure of non-specific psychological distress (Kessler et al., 2003). The K6 assesses the frequency with which respondents experienced symptoms such as hopelessness and worthlessness during the past 12 months. Summed scores of 13 or higher on the K6 indicate serious psychological distress and discriminate highly between individuals with and without a clinical diagnosis of serious mental illness in the general population (Kessler et al., 2003). Respondents with sum scores greater than or equal to 13 in the past year—individuals with probable need for treatment—were selected for analysis.

### Mental Health Treatment

Mental health treatment utilization was assessed using two questions on respondents’ utilization of any mental health services within the past 12 months. Individuals were asked to indicate whether they had received any treatment for “problems with stress, your emotions, or mental health, or for problems with your use of alcohol or drugs” from a mental health specialist (e.g., psychologist; counselor; psychiatrist) or some other type of provider (e.g., general medical provider; chaplain, clergy, or pastor). Items were adapted from the National Survey of Drug Use and Health (NSDUH) (Center for Behavioral Health Statistics & Quality, 2018) and the 2015 HRBS (Meadows et al., 2018).

### Perceived Unmet Treatment Need

Perceived unmet need for mental health treatment was assessed using a NSDUH item that asked whether the respondent felt that that he or she needed mental health care in the past 12 months and did not receive it (yes; no) (Center for Behavioral Health Statistics & Quality, 2018). All individuals with K6 scores ≥ 13 completed this item; note that respondents who endorse this item may have received some care and still thought that they need more or different care (see below).

### Treatment Need Categories

Unmet mental health treatment need among individuals with serious psychological distress was defined in two ways: (1) receiving no mental health services in the past 12 months; and (2) reporting unmet need for mental health services (see above). Based on these data, we defined four mutually exclusive groups of service members, summarized in Table 1. The first group was comprised of individuals with serious psychological distress in the past year who received mental health treatment and did not report any unmet need, which suggests that treatment needs were “met” for these individuals (Group 1: *Treatment Needs Met*). Three additional groups were comprised of individuals with unmet need, as follows: individuals who received mental health treatment and perceive additional unmet treatment need (Group 2: *Treated/Additional Need*); individuals who did not receive mental health treatment and perceive unmet treatment need (Group 3: *Untreated/Recognized Need*); and individuals who did not receive mental health treatment and do not perceive unmet treatment need (Group 4: *Untreated/Unrecognized Need*).

**Table 1:** Treatment need groups among individuals with serious psychological distress

Treatment need group		Any mental health treatment in past year?	Reported unmet treatment need in past year?
*Group 1. Treatedment Needs Met*: Received mental health treatment and did not report any unmet need		Yes	No
*Unmet treatment need groups*			
*Group 2. Treated/Additional Need*: Received mental health treatment and perceive unmet treatment need		Yes	Yes
*Group 3. Untreated/Recognized Need:* Did not receive mental health treatment and perceive unmet treatment need		No	Yes
*Group 4. Untreated/Unrecognized Need:* Did not receive mental health treatment and do not perceive unmet treatment need		No	No

### Barriers to Mental Health Care

**Fig. 1:**
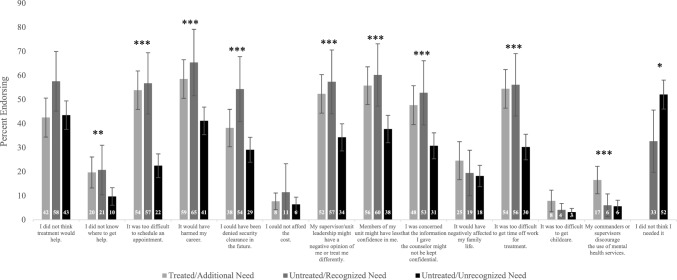
Barriers to treatment endorsed among individuals with unmet treatment need. This figure shows the percentage of individuals endorsing specific barriers to using mental health services within each treatment need group, estimated using analytic weights to account for the sample design and survey response patterns. Numeric percentage values are also shown as labels for each bar. Error bars indicate upper and lower 95% confidence intervals for each estimate. Individuals in the Treated/Additional Need group were coded as missing for “I did not think I needed it”. Significant omnibus group differences in rates of endorsing each barrier, based on bivariate chi-square tests, are indicated as follows: *p = 0.01, **p < 0.01, ***p < 0.001

Reasons for not seeking mental health treatment (barriers to care) were assessed using an item adapted from the NSDUH (Center for Behavioral Health Statistics & Quality, 2018). Respondents were asked to check all reasons that applied to them, and response options covered a range of constructs including awareness of services (“I did not know where to get help”), beliefs about need for treatment (“I did not think I needed it”) and treatment efficacy (“I did not think treatment would help”), stigma and career concerns (e.g., “My supervisor/unit leadership might have a negative opinion of me or treat me differently”), and other factors (see Fig. 1 for a complete list). Individuals who reported perceived unmet treatment need and/or did not receive mental health treatment (i.e., *Treated/Additional Need*, *Untreated/Recognized Need*, and *Untreated/Unrecognized Need* groups) reported on treatment barriers; individuals who endorsed receiving mental health treatment and did not perceive unmet need (*Treatment Needs Met*) did not complete these items. In addition, because they had already engaged in treatment and reported unmet need, individuals in the *Treated/Additional Need* group were coded as missing for “I did not think I needed it.”

### Lost Work

Because functional impairments (e.g., lost work) may constitute an overt sign of a problem (Kahn, 2008) that can enhance perceived need for mental health treatment or other accommodations (Wang et al., 2011), and greater impairment is associated with increased likelihood of treatment use among service members (McKibben et al., 2013), we assessed lost work as another indicator of impairment. Lost work was based on absenteeism and assessed using items modified from the Sheehan Disability Scale (Sheehan et al., 1996), a widely used measure of functional impairment. Lost work days were defined as the number of days of work missed in the past 30 days based on the item: “Did your [mental or physical] symptoms cause you to miss school or work or leave you unable to carry out your normal daily responsibilities? Number of days [0–30].” Due to evidence of skew in the distribution for this variable, we also created a dichotomous indicator to assess rates of more severe impairment, or persistent lost work, defined as 5 or more lost work days in the past month, which corresponds to a full work-week of lost work in the past month.

### Physical Health Comorbidities

Because our assessment of lost work was not specific to mental health symptoms, and physical health conditions may affect lost work/occupational functioning related to mental health problems (France et al., 2012; Merikangas et al., 2007), all models examining associations between lost work and treatment need group control for physical health conditions (hypertension, diabetes/hyperglycemia, high cholesterol, asthma, angina or coronary heart disease, heart attack, back pain, bone, joint, or muscle injury or condition, including arthritis). Physical health conditions were assessed by asking whether respondents had been told in the past 2 years by a doctor or health professional that had any of the following physical health conditions: diabetes, respiratory conditions (including asthma, sinusitis, or chronic bronchitis), arthritis, heart disease or other cardiovascular condition (hypertension, hyperlipidemia), ulcer (digestive system), or any cancer. We also assessed back pain using the back pain item from the commonly-used PHQ-15, a somatic symptom scale (Kroenke et al., 2002). Physical comorbidities were examined as covariates in analyses examining lost work days using separate dummy variables for each condition.

### Demographic and Service Characteristics

Demographic and military service characteristics included: sex, age, race and ethnicity, education level, marital status, service branch, pay grade, deployment history (any deployment [yes/no] and combat deployment [yes/no]).

### Analytic Strategy

All analyses were conducted on the subset of participants with serious psychological distress (K6 score ≥ 13). First, we examined sample descriptive characteristics using weighted univariate statistics (mean [SD] or percent) overall and across the four treatment need groups (see Table 1). We then ran unadjusted bivariate chi-square tests for each variable to examine significant differences in demographic and service characteristics across the four treatment need groups. Next, to examine whether treatment need groups differed with respect to degree or severity of impairment, we use regression models to compare mean differences in psychological distress (K6 scores) across treatment need groups, adjusting for demographic and service characteristics (age, sex, race and ethnicity, education, marital status, service branch, combat deployment, pay grade). We ran similar tests to examine differences in number of lost work days (implemented in linear regression models) and likelihood of persistent lost work (implemented in logistic regression) across treatment need groups, adjusting for demographic and service characteristics as well as comorbid physical health conditions.

Next, to assess differences in self-reported barriers to treatment across the three unmet treatment need groups, we examined the frequency of endorsing each barrier within each group and ran a series of bivariate chi-square tests to examine whether groups differed with respect to rates of endorsing each specific barrier. To account for multiple comparisons and to provide a more conservative assessment of group differences, we only interpret significant findings at *p* ≤ 0.01. All descriptive statistics and regression models used analytic weights to account for the sample design and survey response patterns (i.e., to ensure representative distribution by service branch, pay grade, and gender). Analyses were performed in SAS v 9.4.

## Results

Sample characteristics overall and by treatment need group are detailed in Table 2. Briefly, the sample was 78% male, 85% under age 35, 58% non-Hispanic White, and 23% of participants had some college education or a degree. A majority of the sample was early-career (52% E1-E4), most (59%) had experienced prior deployment, and approximately 39% had history of combat deployment. One quarter of the sample (26%) reported any lost work days in the past month due to a mental and/or physical health condition; average number of lost work days was 2.29 (SD = 0.19). Approximately 61% of participants endorsed any receipt of mental health treatment within the past 12 months, and one quarter (25%) reported any unmet treatment need in the past year.

**Table 2:** Characteristics of service members with current serious psychological distress in the 2018 HRBS and differences by treatment need group

		Unmet need groups			Overall group difference (*p *value)
	Treatment needs met	*Treated/ additional need*	*Untreated/ recognized need*	*Untreated/ unrecognized need*	
	n = 1,076 (43.05%)	n = 429 (17.54%)	n = 147 (7.29%)	n = 684 (32.11%)	
	Percent (95% CI)/Mean(SD)	Percent (95% CI)/Mean(SD)	Percent (95% CI)/Mean(SD)	Percent (95% CI)/Mean(SD)	
Sex					
Female	24.39 (21.14–27.63)	25.80 (20.35–31.25)	19.63 (11.29–27.96)	17.83 (14.37–21.30)	*p* = 0.03
Male	75.61 (72.37–78.86)	74.20 (68.75–79.66)	80.37 (72.04–88.71)	82.17 (78.70–85.63)	
Age group					
18–24	40.10 (34.80–45.39)	38.81 (30.19–47.44)	41.19 (28.37–54.01)	53.91 (48.13–59.69)	***p* = 0.0002**
25–34	40.46 (35.63–45.09)	40.99 (33.37–48.61)	46.65 (32.98–60.31)	34.83 (29.63–40.04)	
35–44	16.73 (13.47–19.99)	17.87 (13.66–22.08)	11.12 (6.23–16.02)	9.95 (7.90–12.01)	
45+	2.82 (2.04–3.59)	2.33 (1.36–3.31)	1.04 (0.21–1.88)	1.31 (0.43–2.18)	
Race and ethnicity					
Non-Hispanic White	57.93 (53.11–62.75)	62.85 (55.23–70.46)	67.21 (54.78–79.63)	54.74 (48.80–60.67)	*p* = 0.02
Non-Hispanic Asian	5.86 (3.85–7.87)	2.94 (0.91–4.97)	2.50 (0.33–4.67)	7.49 (4.91–10.07)	
Non-Hispanic Black	18.62 (14.99–22.25)	14.78 (9.30–20.25)	18.60 (6.62–30.57)	13.05 (9.22–16.87)	
Hispanic	14.30 (10.67–17.93)	13.76 (9.29–18.23)	5.45 (2.04–8.86)	19.68 (14.70–24.65)	
Non-Hispanic Other	3.29 (1.95–4.62)	5.68 (0.31–11.04)	6.25 (1.72–10.79)	5.05 (3.14–6.97)	
Education					
High School or less	73.80 (70.37–77.23)	75.34 (70.26–80.43)	82.62 (75.99–89.26)	80.36 (76.74–83.97)	*p* = 0.04
Some College	12.95 (10.46–15.43)	12.20 (8.67–15.72)	7.09 (2.72–11.46)	8.28 (5.63–10.94)	
College Degree	13.25 (11.01–15.49)	12.46 (9.21–15.70)	10.29 (5.77–14.81)	11.36 (8.96–13.76)	
Marital status					
Married	45.83 (40.91–50.76)	50.19 (42.17–58.22)	45.06 (31.65–58.46)	44.36 (38.45–50.28)	***p* = 0.003**
Cohabiting	8.35 (5.39–11.30)	8.95 (5.30–12.60)	11.33 (3.37–19.29)	11.15 (7.05–15.25)	
Separated, Divorced, or Widowed	15.48 (11.45–19.51)	10.02 (5.64–14.41)	8.09 (3.53–12.64)	4.96 (3.07–6.85)	
Never Married	30.24 (25.46–35.23)	30.84 (22.48–39.20)	35.53 (22.36–48.70)	39.52 (33.56–45.48)	
Ever deployed (% yes)	57.97 (52.83–63.10)	58.50 (50.25–66.76)	60.85 (47.54–74.16)	60.37 (54.46–66.39)	*p* = 0.93
Deployed within past 12 months (% yes)	26.84 (22.16–31.52)	23.88 (17.13–30.63)	35.11 (22.73–47.49)	32.32 (26.63–38.01)	*p* = 0.17
Ever combat deployed (% yes)	40.18 (35.48–44.89)	45.98 (38.22–53.73)	27.01 (16.80–37.21)	37.83 (32.23–43.44)	*p* = 0.05
Pay grade					
E1–E4	50.86 (45.86–55.87)	46.40 (38.13–54.66)	57.40 (45.06–69.75)	58.41 (52.90–63.92)	*p* = 0.02
E5–E6	30.44 (26.35–34.53)	34.45 (27.34–41.56)	30.01 (19.19–40.83)	25.51 (20.82–30.20)	
E7–W5	10.65 (7.70–13.61)	10.64 (7.65–13.63)	5.77 (2.74–8.79)	6.68 (4.98–8.38)	
O1–O3	5.43 (4.14–6.72)	5.90 (3.57–8.22)	4.30 (1.32–7.28)	7.39 (5.44–9.34)	
O4–O6	2.62 (1.96–3.28)	2.61 (1.55–3.66)	2.52 (0.77–4.27)	2.02 (1.32–2.71)	
Service branch					
Air Force	16.03 (13.63–18.42)	12.40 (9.19–15.60)	10.73 (6.02–15.44)	14.00 (11.31–16.69)	*p* = 0.07
Army	37.64 (32.29–42.98)	41.74 (33.52–49.96)	33.76 (19.59–47.93)	30.13 (23.90–36.36)	
Marine Corps	16.93 (13.43–20.42)	11.71 (7.82–15.60)	15.79 (8.27–23.31)	18.83 (14.65–23.01)	
Navy	27.43 (23.07–31.78)	32.47 (24.68–40.26)	37.43 (24.40–50.46)	34.76 (28.98–40.55)	
U.S. Coast Guard	1.98 (1.42–2.54)	1.69 (0.95–2.43)	2.28 (0.64–3.92)	2.28 (1.44–3.12)	

Full sample N = 2,336. Percentages are estimated using analytic weights to account for the sample design and survey response patterns. Omnibus group differences were assessed using bivariate chi-square tests. Bolded values indicate statistically significant group differences at *p* ≤ 0.01

With respect to treatment need groups, 43% of individuals received treatment and did not endorse any unmet needs (*Treatment Needs Met*). Approximately 18% received treatment but also endorsed unmet need (*Treated/Additional Need),* approximately 7% had no treatment and endorsed unmet need (*Untreated/Recognized Need),* and nearly a third of the sample (32%) had no treatment and no perceived unmet need (*Untreated/Unrecognized Need*) (see Table 2).

As shown in Table 2, treatment need groups differed with respect to some demographic and service characteristics, but few differences were significant at *p* ≤ 0.01. Groups differed significantly with respect to age group composition (${\rm X}_{{\text{DF}} = {9}}^{2}$ = 32.70, *p* = 0.0002) and marital status (${\rm X}_{{\text{DF}} = {9}}^{2}$ = 25.30, *p* = 0.003) such that those in the *Untreated/Unrecognized Need* group had the highest proportion of individuals ages 18–24 and the highest rates of endorsing “never married.”

Table 3 shows differences across treatment need groups with respect to degree of impairment indicated by psychological distress and lost work days. Adjusting for demographic and service characteristics, groups differed with respect to psychological distress, such that individuals in the *Treated/Additional Need* group had slightly but statistically significantly higher (*p* < 0.001) K6 scores compared to individuals in the *Treatment Needs Met* and *Untreated/Unrecognized Need* groups. In addition, adjusting for demographic and service characteristics and physical health comorbidities, individuals in the *Treated/Additional Need* group reported a greater number of lost work days compared to those in the *Treatment Needs Met* and *Untreated/Unrecognized Need* groups (see Table 3). We observed a similar pattern with respect to likelihood of reporting persistent lost work (i.e., 5 or more days in the past month).

**Table 3:** Differences in degree of psychological distress and lost work by treatment need group

		*Unmet need groups*			Overall group difference (*p *value)
	Treatment needs met	*Treated/ Additional Need*	*Untreated/ Recognized Need*	*Untreated/ Unrecognized Need*	
	n = 1,076	n = 429	n = 147	n = 684	
	Mean (95% CI)/ Percent (95% CI)	Mean (95% CI)/ Percent (95% CI)	Mean (95% CI)/ Percent (95% CI)	Mean (95% CI)/ Percent (95% CI)	
Psychological distress severity (K6 score)^1^	17.98 (17.76–18.19)	18.77 (18.43–19.10)^a^	17.22 (16.71–17.74)^b^	17.33 (17.08–17.58)^a,b^	*p* < 0.0001
Number of lost work days^2^	2.26 (1.89–2.64)	4.05 (3.47–4.64)^a^	1.63 (0.74–2.53)^b^	1.58 (1.15–2.01)^b^	*p* < 0.0001
Percent reporting persistent lost work (5 + days in the past month)^3^	12.18 (9.38–15.66)	21.23 (15.55–28.28)^a^	9.57 (3.63–22.91)	8.01 (5.43–11.67)^b^	*p* = 0.0009

All models used analytic weights to account for the sample design and survey response patterns

^1^Values are model-based least square means from linear regression models adjusting for service branch, sex, paygrade, age, education, race, marital status, and combat deployment history

^2^Values are model-based least square means from linear regression models adjusting for service branch, sex, paygrade, age, education, race, marital status, combat deployment history, and physical health comorbidities

^3^Values are model-based percentages from logistic regression models adjusting for service branch, sex, paygrade, age, education, race, marital status, combat deployment history, and physical health comorbidities

^a^ Indicates statistically significant difference in least square means at *p* < 0.01 from pairwise comparison to *Treatment Needs Met* group

^b^ Indicates statistically significant difference in least square means at *p* < 0.01 from pairwise comparison to *Treated/Additional Need* group

Across all individuals with unmet need (n = 1,260), the most commonly endorsed barriers included concerns surrounding potential harms to one’s career (50%), beliefs that members of one’s unit would have less confidence in them (46%), and beliefs that treatment would not help (45%). Approximately one third (34%) of individuals endorsed the belief that they did not need treatment. The least commonly endorsed treatment barriers pertained to commanders/supervisors discouraging use of mental health care (9%), concerns about treatment costs (7%), and difficulties related to child care (5%).

Figure 1 shows patterns of endorsing specific barriers to accessing treatment across the three groups with unmet treatment need, by subgroup. Bivariate omnibus chi-square tests indicated that the three unmet need groups differed significantly in rates of endorsing several barriers (see Fig. 1). As shown in Fig. 1, patterns of endorsing barriers were largely similar across the groups that self-reported unmet need– *Treated/Additional Need* and *Untreated/Recognized Need*. In these groups, the most commonly endorsed barrier to seeking care was “It would have harmed my career,” endorsed by 59% of individuals in the *Treated/Additional Need* group and 65% of those in the *Untreated/Recognized Need* group. Although “My commanders or supervisors discourage the use of mental health services" was among the least-commonly endorsed barriers, groups differed with respect to this item (${\rm X}_{{\text{DF}} = 1}^{2}$ = 19.27, *p* < 0.0001) such that those in the *Treated/Additional Need* group showed the highest rate of endorsement (17%) compared to the *Untreated/Recognized Need* (6%) and *Untreated/Unrecognized Need* (6%) groups.

Individuals in the *Untreated/Unrecognized Need* group showed systematically lower rates of endorsing most barriers relative to the other two groups. The most commonly endorsed barriers among the *Untreated/Unrecognized Need* group were beliefs that treatment would not help (43%) and beliefs that they did not need treatment (52%). Those in the *Untreated/Unrecognized Need* group were more likely to endorse beliefs that they did not need treatment (${\rm X}_{{\text{DF}} = 1}^{2}$ = 18.91, *p* = 0.01) compared to those in the *Untreated/Recognized Need* group (i.e. partially validating their treatment need group label). Groups did not differ significantly with respect to beliefs that treatment would not help, concerns about cost, concerns about effects on family life, and child care difficulties.

## Discussion

Among this sample of U.S. service members with probable need for treatment, more than half of respondents met study criteria for unmet mental health treatment need. This is consistent with past work showing that unmet treatment need is a common and serious problem among active service members (Fikretoglu et al., 2022; Hom et al., 2017). Nearly one third of the sample did not receive mental health treatment and did not perceive unmet need, indicating that a substantial proportion of service members who could benefit from mental health treatment do not recognize a need for care. Moreover, approximately one in five individuals with past-year distress had received mental health care but reported remaining unmet need, perhaps for different or better care. A minority of respondents (7% of this high need sample) reported no mental health treatment in the past year *and* acknowledged a need for care that was not being met. Effective approaches to meeting the needs of these subgroups may be very different, and understanding how groups differ with respect to military and demographic characteristics and unique barriers to care has implications for understanding patterns of proactive self-referral for services (Ghahramanlou-Holloway et al., 2019; Rowan et al., 2014) and for tailoring strategies to address mental health issues for individuals with different need profiles. Overall, our findings are consistent with prior studies and suggest that barriers to engaging in services may differ across service members depending on recognition of needs and their stage in the help-seeking process (i.e., whether or not they have already engaged in treatment) (Fikretoglu et al., 2022).

The four treatment need groups examined in this study differed with respect to level of impairment. For example, individuals in the *Untreated/Unrecognized Need* group showed slightly lower levels of impairment with respect to lost work days and severity of symptoms on the K6 compared to individuals who had engaged in treatment and perceived additional need. This may suggest that, despite reporting relatively severe symptoms consistent with the presence of a mental health condition, symptoms may not have yet resulted in severe enough impairments to prompt care seeking (McKibben et al., 2013; Murphy et al., 2014) in this group. Consistent with this, the most commonly reported ‘barrier’ to seeking care among individuals in the *Untreated/Unrecognized Need* group was feeling as though they did not need treatment (endorsed by over 50% of these individuals); the next most commonly endorsed barrier was believing that treatment would not help. In the absence of appropriate treatment, mental health symptoms may persist or worsen and lead to more significant functional impairments over time. As a whole, this pattern of findings may suggest that knowledge-focused interventions, such as awareness campaigns (Acosta et al., 2020), efforts to clarify point-of-access and processes for engaging in care (Rafferty et al., 2020), and programs targeting mental health literacy (Thomas et al., 2016) could be helpful in reducing unmet treatment need for service members who may not recognize signs or symptoms of a mental health problem. Of note, evidence for the impact of mental health literacy trainings on improving engagement in services and treatment outcomes—for both military and civilian populations—is limited (Fikretoglu et al., 2022; Forthal et al., 2022; Gulliver et al., 2012; Thomas et al., 2016). In recent years, a handful of studies have reported on other evidence-based programs (e.g., unit group-based training to support solders with mental health conditions) (Britt et al., 2018) that have shown promise in enhancing help-seeking in samples of military personnel (Fikretoglu et al., 2022). More rigorous research is needed to assess the longitudinal impact, sustainability, and scalability of evidence-based interventions to address service members’ mental health needs.

Among those who reported unmet treatment need, it was more common for individuals to report receiving some mental health treatment in the past year than to report no treatment. This suggests that most self-reported need in the sample reflected a need for additional or different care beyond what individuals had already received (although note: this study did not assess specific types or sources of care accessed). However, there were few systematic differences with respect to individual characteristics across the *Treated/Additional Need* and *Untreated/Unmet Need* groups, and both groups were systematically more likely to endorse a range of barriers to accessing care—at similar rates—compared to those who did not recognize a need for care. Beliefs that treatment would not help and barriers related to stigma and the impact of treatment-seeking on one’s career were among the most commonly endorsed reasons for not seeking treatment in these groups, which is consistent with prior reports of barriers to care-seeking in military samples (Britt et al., 2016; Fikretoglu et al., 2022; Ghahramanlou-Holloway et al., 2019; Hom et al., 2017; Kim et al., 2011; Momen et al., 2012; Sharp et al., 2015; Zinzow et al., 2013). For individuals who may have already recognized their symptoms as a problem, beliefs about potential negative consequences of seeking help may be a key barrier to engaging in and/or receiving optimal care (Stevelink et al., 2019). Military culture places a high value on personal strength and competence, which may act to reinforce stigma surrounding help-seeking and preferences for self-management of mental health problems among active duty service members (Adler et al., 2015; Langston et al., 2007; Pury et al., 2013). Programs targeting stigmatizing attitudes may be particularly important for those who already recognize need for treatment (Britt et al., 2016; Rafferty et al., 2020). However, evidence for the effects of stigma reduction interventions on service utilization among military populations is quite limited (Sharp et al., 2015). More research on whether and how such interventions may affect treatment engagement among different groups of service members is warranted (Fikretoglu et al., 2022). It is also notable that in this sample, as in other studies (Hom et al., 2017), endorsement of commander/supervisor discouragement of mental health service use was relatively low (9%)—but, it was not zero; nor was it the least commonly-reported barrier. In addition, rates of endorsing this barrier were significantly higher in the group that had already engaged in treatment and perceived additional unmet need (17% endorsed) compared to other groups. Leadership can play an important role in modeling appropriate service utilization and positive mental health practices (Britt et al., 2018; Kim et al., 2011; Zinzow et al., 2013). As such, continuing efforts to support leaders in this role could help to reduce unmet treatment need.

In contrast to the civilian population in the U.S., military personnel have universal access to high quality, low cost mental health services. In conjunction with widespread screening programs already implemented by the military, this presents a unique opportunity to address unmet mental health treatment need. The Department of Defense invests considerable resources into assessing service members’ mental health and implementing prevention programs to help mitigate the negative impacts of mental health conditions on service member wellbeing. Findings from this study suggest a need to continue such efforts, and study their effects, in order to address unmet need across the full spectrum of service members with treatment needs, which includes a sizeable portion of individuals who may not recognize a need for treatment.

These findings should be considered in the context of several limitations. First, data were cross-sectional, which limits the extent to which causal relationships can be inferred from these analyses. In addition, the assessment of lost work used in the HRBS is not specific to mental health conditions. Although we controlled for a range of physical health conditions, along with other individual characteristics, we cannot directly attribute work impairments to mental health symptoms. We also did not examine types of treatment or other sources of support (e.g., informal peer support) that individuals may have sought out, which may differ in relation to mental health symptoms and other service member characteristics (Stevelink et al., 2019). Additional research assessing different sources of support, recognition of need, and barriers could yield important insights into ways to reduce unmet need. Moreover, we did not examine differences in relation to other factors that may influence recognition of need and engagement in treatment, such as alcohol and other substance use (Stevelink et al., 2019). Furthermore, response rates to the 2018 HRBS were low. Although all data were weighted to account for non-response bias and to help ensure representativeness of data to the broader active duty U.S. military population, we cannot rule out the possibility that respondents may have been systematically different from non-respondents on some characteristics.

In conclusion, this study extends the evidence base on unmet treatment need and barriers to treatment use by describing differences across subgroups of service members with high need for mental health treatment. Among those with unmet treatment need, the largest subgroup was comprised of individuals who had not received care and did not recognize a need for treatment. Moreover, findings suggest that individuals who *perceive* unmet need systematically differ from those who do not with respect to treatment barriers. These differences have implications for reducing unmet treatment need among all service members who may benefit from treatment, including those who may not yet perceive a need for services and those who have already taken the step to engage in treatment and have additional needs. Continued use of diverse strategies, including efforts to enhance mental health literacy and address common barriers to accessing care (e.g., stigma, career concerns), may be important for enhancing treatment utilization and reducing unmet need across the full spectrum of individuals who may benefit from care.
