# Correction to: Heterogeneity in Unmet Treatment Need and Barriers to Accessing Mental Health Services Among U.S. Military Service Members with Serious Psychological Distress

**DOI:** 10.1007/s10488-024-01379-x

**Published:** 2024-05-04

**Authors:** Michael S. Dunbar, Joshua Breslau, Rebecca Collins, Robin Beckman, Charles C. Engel

**Affiliations:** 1 RAND Corporation, 4750 Fifth Avenue, Suite 600, Santa Monica, PA 15213-2665 USA; 2 RAND Corporation, 1776 Main Street, Santa Monica, CA 90407 USA; 3 Department of Psychiatry and Behavioral Sciences, University of Washington, 1959 NE Pacifc Street, Seattle, WA 98195 USA; 4 Health Services Research & Development Center for Innovation, VA Puget Sound Health Care System, 1660 S. Columbian Way, Seattle, WA 98108 USA

**Correction to: Administration and Policy in Mental Health and Mental Health Services Research**

10.1007/s10488-023-01289-4

The copyright holder for this article was incorrectly given as “The Author(s)” 2023 but should have been “RAND Corporation” 2023.

The original article has been corrected.

